# Fast and precise detection of DNA methylation with tetramethylammonium-filled nanopore

**DOI:** 10.1038/s41598-017-00317-2

**Published:** 2017-03-15

**Authors:** Ying Wang, Yani Zhang, Yanli Guo, Xiao-feng Kang

**Affiliations:** 1 Key Laboratory of Synthetic and Natural Functional Molecular Chemistry, College of Chemistry & Materials Science, Northwest University, Xi’an, 710069 P. R. China; 2 College of Life Sciences, Northwest University, Xi’an, 710069 P. R. China

## Abstract

The tremendous demand for detecting methylated DNA has stimulated intensive studies on developing fast single-molecule techniques with excellent sensitivity, reliability, and selectivity. However, most of these methods cannot directly detect DNA methylation at single-molecule level, which need either special recognizing elements or chemical modification of DNA. Here, we report a tetramethylammonium-based nanopore (termed TMA-NP) sensor that can quickly and accurately detect locus-specific DNA methylation, without bisulfite conversion, chemical modification or enzyme amplification. In the TMA-NP sensor, TMA-Cl is utilized as a nanopore-filling electrolyte to record the ion current change in a single nanopore triggered by methylated DNA translocation through the pore. Because of its methyl-philic nature, TMA can insert into the methylcytosine-guanine (^m^C-G) bond and then effectively unfasten and reduce the ^m^C-G strength by 2.24 times. Simultaneously, TMA can increase the stability of A-T to the same level as C-G. The abilities of TMA (removing the base pair composition dependence of DNA strands, yet highly sensing for methylated base sites) endow the TMA-NP sensor with high selectivity and high precision. Using nanopore to detect dsDNA stability, the methylated and unmethylated bases are easily distinguished. This simple single-molecule technique should be applicable to the rapid analysis in epigenetic research.

## Introduction

DNA methylation at cytosine residues (5 ^m^Cs) is a well-known epigenetic marker that plays a critical role in the regulation and maintenance of many important cellular processes, including genomic imprinting^1^, embryonic development^2^, transcription^3^, chromatin structure^4^, X chromosome inactivation^5, 6^, and chromosome stability^7^. As a form of the most common epigenetic change, 5 ^m^C often occurs in CpG dinucleotides (5′-cytosine-phosphate-guanine-3′), particularly in CpG islands of regulatory regions. CpG islands, which contain tens to hundreds of CpG repetitions, are the major component of the promoter regions of housekeeping genes or other genes frequently expressed in a cell^8, 9^. The presence of methylated CpG sites in CpG islands of promoters causes their downstream genes, such as tumour suppressor genes, to be consistently silenced^10–12^. In the human genome, aberrant DNA methylation has been implicated in many diseases such as diverse cancers, in which gross hypomethylation is accompanied by hypermethylation within certain CpG islands^13^. In clinical medicine, some drugs have been used in epigenetic therapy by reactivating silenced genes, such as 5-aza-2ʹ-deoxycytidine (5-aza) for methylation-related azanucleoside disease^14^. Unlike DNA sequences, methylation patterns may vary at various stages of therapy, even between cells^14, 15^, which requires fast, inexpensive, and reliable detection methods for small, native, unamplified DNA samples. Hence, the analysis of methylated DNA as a potential biomarker is increasingly important in basic studies and molecular diagnostics for cancer risk assessment, early diagnosis, prognosis and epigenetic therapy.

Currently, most of the available bulk techniques for detecting DNA methylation rely on bisulfite conversion (converting unmodified cytosine to uracil in DNA), followed by various sequencing techniques, such as bisulfite sequencing^16–18^, methylation-specific PCR^19^, bisulfite pyrosequencing^20^, combined bisulfite restriction analysis assay^21^, the MethyLight assay^22^, mass spectrometry detection^23^ and Illumina BeadArray^24^. These bisulfite conversion-based methods can map the whole-genome, but the expensive DNA degradation, DNA damage and incomplete conversion during bisulfite conversion render them unsuitable for reliable and precise locus-specific analysis. Some new assays have been reported that do not require bisulfite conversion. Methylation-specific enzyme restriction can provide information on broadly spaced 5 ^m^Cs by the recognition of protein enzymes and subsequent sequencing, but does not work for densely packed 5 ^m^Cs^25^. Labelling methylcytosine with antibodies, methyl-CpG binding proteins, and metal complexes such as osmium or vanadium complexes are suitable for bulk assays, but those assays require large samples and lack sensitivity at single-base resolution; thymine is also unfortunately labelled via OsO_4_ in addition to methylcytosine^26–32^. DNA methylation has also been analysed by capillary electrophoresis (CE)^33, 34^. CE-coupled laser-induced fluorescence (LIF) analysis provides good sensitivity and uses small DNA samples. However, the CE method is time-consuming and laborious, requiring DNA digestion followed by chemical derivatization of the mononucleotides^34^. Furthermore, many methods either require expensive instruments or are laborious, involving chemical modification and amplification. These restrict their applications in clinical analysis.

Nanopore technology as a single-molecule detection tool is attractive for DNA analysis because of its inherent merits such as speed, simplicity, low cost, and label-free and amplification-free detection^35–41^. However, the protein nanopore sensor itself fails to distinguish methylated DNA from unmethylated DNA in the simple salts that are traditionally used in nanopore stochastic sensing, such as KCl or NaCl^42–48^. To achieve this goal, nanopores have required special recognizing elements; alternately, methylated DNA has been chemically converted in some strategies. Most of these methods cannot directly detect DNA methylation without special recognizing elements or chemical modification of DNA at single-molecule level. The phi29 DNA polymerase motor-coupled MspA nanopore can pull ssDNA molecules through the pore in single-nucleotide steps and map ^m^C with single-nucleotide resolution, but the loose and leaky junction (non-alignment) between polymerase and pore resulted in interruption and failure of detection^42, 43^. The combination of bisulfite conversion with a uracil-Hg^2+^-thymine complex, with no inbuilt measure of adequacy of the bisulfite treatment, has the possibility of false positives because of potential inadequate conversion of non-methylated cytosine to uracil^44^. Methylated CpG dinucleotides in DNA have been labelled with methyl DNA binding protein (MBD), streptavidin or a ferrocene ⊂ cucurbit uril complex to produce an observable or highly characteristic ion current blockade, but the bulkiness of the conjugated proteins may result in a failure to detect densely methylated regions in the genome, and multiple orientations of attached molecules in the nanopore could also result in a loss of reproducibility^46–48^. Accordingly, the development of a novel single-molecule biosensor that can quickly and accurately detect 5 ^m^Cs is an urgent and important need.

TMA^+^ is the simplest quaternary ammonium cation, consisting of four methyl groups attached to a central nitrogen atom. TMA salt is often used as a supporting electrolyte in organic electrochemistry. We previously reported that TMA-Cl could be used as a pore-filling electrolyte to improve single-nanopore recording abilities, such as elevating the stability of the planar lipid bilayer, reducing recording noise, and extending single-channel lifetime^49^. Previous work has also shown that polymer-cation binding leads to an increase in the affinity between the polymer and the pore, thus controlling the residence time of the polymer in the pore^39^. In this communication, We found that in TMA-Cl, the stability of methylated DNA hairpin relative to unmethylated DNA is significantly reduced, which might be due to TMA insertion into the ^m^C-G base pairs in CpG. Simultaneously, TMA can eliminate the stability difference of A-T vs G-C base pairs of DNA hairpin in nanopore analysis. Using these characteristics, we have developed a single-molecule TMA-NP method for identifying and detecting unmethylated and methylated DNA through a universal probe.

## Results

### Differentiation of methylated and unmethylated DNA

**Figure 1:**
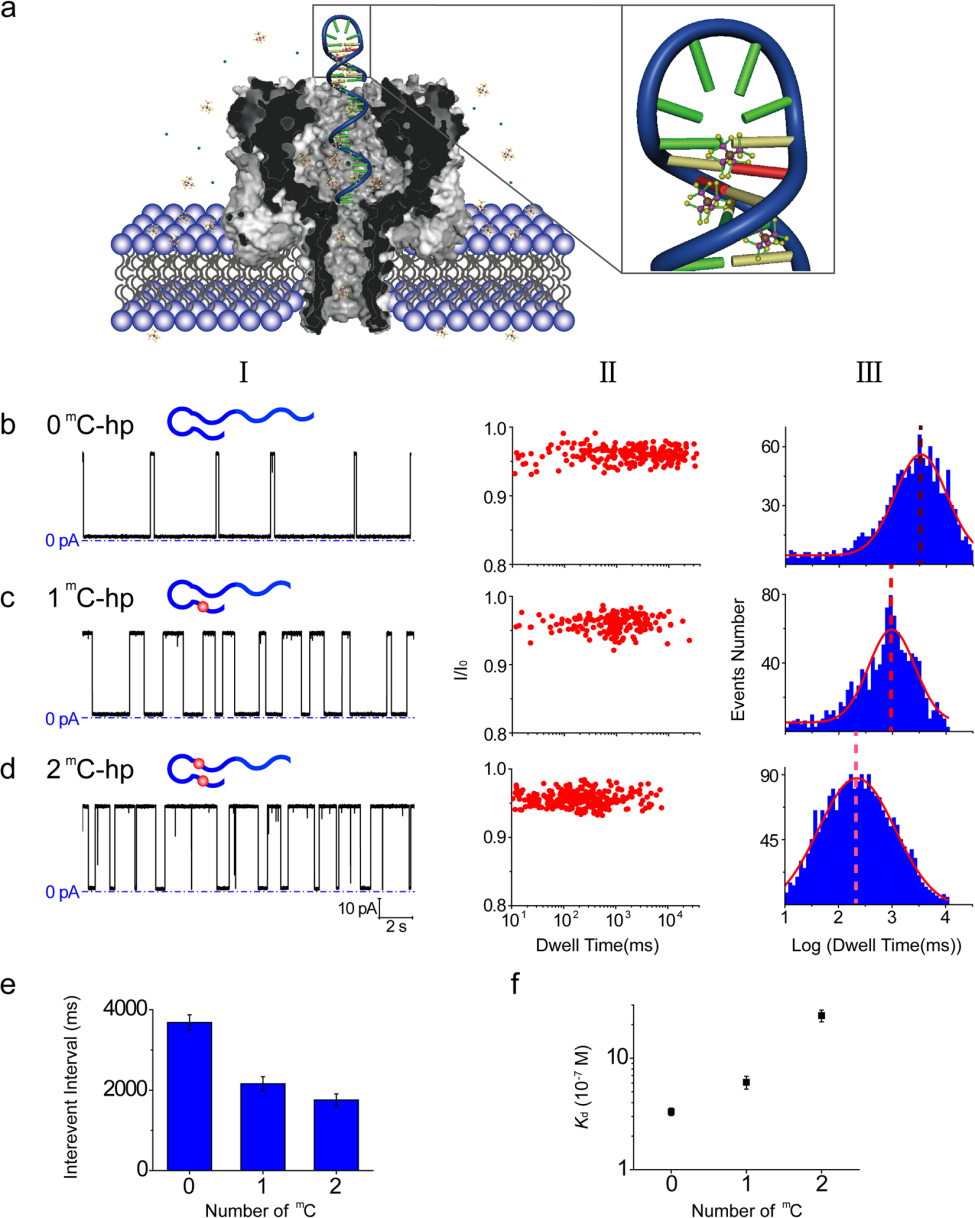
TMA-NP discrimination of unmethylated and methylated DNA. (**a**) Schematic of the methylated DNA hairpin containing two 5′-methylcytosines (red) at duplex (2 ^m^C-hp) translocating through the wild-type α-hemolysin (α-HL) nanopore in 4 M TMA-Cl. (**b**–**d**) Representative single-channel recording traces (column I), scatter plot of I/I_0_ vs dwell time (column II) and dwell time histogram in log form (column III) for unmethylated DNA (0 ^m^C-hp) (**b**), methylated DNA containing one 5′-methylcytosine (1 ^m^C-hp) (**c**) and two 5′-methylcytosines (2 ^m^C-hp) (**d**) in 4 M TMA-Cl. 5′-Methylcytosine is shown as red circles. The signals were filtered at 2 kHz and sampled at 20 kHz. Scatter plot and dwell time histograms are collected from 300 events. I/I_0_ is normalized blockage current, which was obtained by dividing the average blockage current of an event by the average open channel current. The distribution of the dwell time histogram was fitted to a Gaussian function. (**e**,**f**) Mean interevent interval (**e**) and equilibrium dissociation constant (*K*_d_) (**f**) for 0 ^m^C-hp, 1 ^m^C-hp and 2 ^m^C-hp. The interevent interval values were obtained from the interevent interval histograms by fitting the distributions to single exponential functions. Data points were averaged with sd from n = 6 experiments. The recordings were made at 25 °C, and the applied potential was +40 mV (a positive potential at the *trans* side and the *cis* side at ground). DNA was added into the *cis* chamber with a final concentration of 0.3 µM. The buffer was 10 mM Tris-HCl (pH 8.5).

A single TMA-filled nanopore (TMA-NP) was used as a single-molecule discriminator for DNA methylation. To test the differentiation ability of TMA-NP between methylated and unmethylated DNA, we chose a 38-nt DNA hairpin fragment as unmethylated DNA (0 ^m^C-hp), which can be directly measured using the α-HL nanopore by lodging the hairpins in the α-HL vestibule^50^. 0 ^m^C-hp contained a 20 cytosine (C)-base “tail” to pull the DNA hairpin through the nanopore, a 4 C-base loop and a 7-base-pair alternating C-G stem to avoid irrelevant forces (Fig. 1a and Table S1). In the DNA design, partial hairpin and loop structures were used to obtain relatively simple and subtle nanopore signals, considering both the fact that TMA-Cl would preferentially interact with A-T base pairs^51^, and the self-hybridization of poly (dG)^52, 53^. The methylated DNA adopted the same sequence as the unmethylated DNA (0 ^m^C-hp), except for one 5′-methylcytosine (1 ^m^C-hp) or two 5′-methylcytosines (2 ^m^C-hp) at the hairpin duplex, respectively (Table S1). The electrophoretic transport of a DNA fragment through a wild-type α-hemolysin (WT α-HL) nanopore is schematically illustrated in Fig. 1a. The internal constriction diameter of the pore (1.4 nm) is larger than the diameter of ssDNA (1.2 nm) and less than the diameter of dsDNA (2.2 nm)^54^. As the DNA is driven into the pore from the *cis* entrance in an electrical field, the tail enters into the *β*-barrel first and the duplex domain is trapped in the vestibule, and then the duplex domain unzips and translocates through the nanopore by electric field force^55, 56^.

The typical current traces for 0 ^m^C-hp, 1 ^m^C-hp and 2 ^m^C-hp passing through the α-HL nanopore are shown in Fig. 1b–d. The methylated hairpin unzipped and translocated through the nanopore at a higher speed than the unmethylated hairpin. The log-form histograms of dwell time for 0 ^m^C-hp, 1 ^m^C-hp and 2 ^m^C-hp peaked at 3.50 ± 0.02 (3162 ± 149 ms) (mean ± s.d.), 2.99 ± 0.02 (977 ± 46 ms) and 2.27 ± 0.02 (186 ± 8 ms), n = 6, respectively. The translocation speed (1/dwell time) for 1 ^m^C-hp and 2 ^m^C-hp increased 3.24 times and 17 times compared with that for 0 ^m^C-hp. The results suggest that DNA methylation greatly destabilizes the duplex in the TMA-NP system and results in shortened unzipping time. Normally, the ^m^C-G bond is stronger than the C-G bond^57^. The remarkable decrease of ^m^C-G stability can be ascribed to the insertion of TMA into the methylcytosine-guanine (^m^C-G) bond.

Figure S1 displays the voltage dependencies of the mean dwell times for unmethylated and methylated DNA, which were fitted from the dwell time histogram to exponential functions. The datasets demonstrated that the dwell time values for 0 ^m^C-hp, 1 ^m^C-hp and 2 ^m^C-hp decreased at least two orders of magnitude with an applied voltage shift from +40 mV to +120 mV: 3388 ± 160 ms to 6.3 ± 0.3 ms (0 ^m^C-hp), 1047 ± 49 ms to 4.7 ± 0.9 ms (1 ^m^C-hp) and 219 ± 6 ms to 3.6 ± 0.6 ms (2 ^m^C-hp). The higher voltage resulted in larger electrophoretic force, thus producing shorter dwell time. According to the Kramers rate model^58^ and the Meller nanopore model^55^, the observed exponential dependence of dwell time on the applied voltage demonstrates that the DNA fragments driven by the voltage enter into the pore from the cis side and assuredly exit from the trans side. By the data fitted to the model equation of dwell time ${\tau }_{{\rm{off}}}=A{e}^{({E}_{{\rm{b}}}-{Q}_{{\rm{eff}}}V)/{k}_{{\rm{B}}}T}$ (detail, see S1), the zero voltage transition time *τ*_0_ $({\tau }_{0}=A{e}^{{E}_{{\rm{b}}}/{k}_{{\rm{B}}}T})$ was derived: 7.79 × 10^4^ ms (0 ^m^C-hp), 1.66 × 10^4^ ms (1 ^m^C-hp) and 2.45 × 10^3^ ms (2 ^m^C-hp). Comparing the energy barriers (*E*_b_) of 0 ^m^C-hp to those of 1 ^m^C-hp and 2 ^m^C-hp produced two energy differences of 1.55 J and 2.05 J, respectively, displaying the ability of DNA methylation to reduce the energy barrier for dissociation of the hairpin. -*Q*_eff_ *V* is the reduction in the energy barrier due to the electric field. From the *Q*_eff_/*k*_B_ *T* value, we estimate the effective charge (*Q*_eff_) in the pore: *Q*_eff_ (0 ^m^C-hp) = 2.03*e*, *Q*_eff_ (1 ^m^C-hp) = 1.77*e* and *Q*_eff_ (2 ^m^C-hp) = 1.54*e*. These effective charge values further verify the insertion of TMA at the methylcytosine point. Moreover, the hairpin stability with increasing ^m^C bases showed the same tendency at any voltage: 0 ^m^C-hp > 1 ^m^C-hp > 2 ^m^C-hp. We therefore can identify methylated DNA at any voltage from +40 mV to +120 mV. However, at +40 mV, the most distinct difference was observed, which was very favourable for discriminating methylated modification.

The interevent interval (unoccupied state of nanopore, *τ*_on_) also increased with DNA methylation (See Fig. 1e). Compared with 0 ^m^C-hp (3758 ± 191 ms), the interevent interval values for 1 ^m^C-hp (2216 ± 167 ms) and 2 ^m^C-hp (1657 ± 150 ms) were decreased 0.41-fold and 0.56-fold, respectively. We further compared dissociation constants (*K*_d_). Using *τ*_on_ and *τ*_off_ values, association (*k*_on_ = 1/c · *τ*_on_) and dissociation (*k*_off_ = 1/*τ*_off_) rate constants for 0 ^m^C-hp, 1 ^m^C-hp and 2 ^m^C-hp are derived and further yield the corresponding *K*_d_ values (*K*_d_ = *k*_off_/*k*_on_) and Gibbs free energy (*Δ*G = −RTln(1/*K*_d_)), where R is the universal gas constant with a value of 8.314 J · K^−1^ · mol^−1^ and T is absolute temperature (Table S2). At a DNA concentration of c = 0.3 µM, the *K*_d_ and *Δ*G values for 0 ^m^C-hp are (3.31 ± 0.36) × 10^−7^ M and −37.0 ± 0.3 kJ · mol^−1^. The *K*_d_ values for 1 ^m^C-hp and 2 ^m^C-hp increase 1.92 times and 6.86 times to (6.37 ± 0.80) × 10^−7^ M and (2.27 ± 0.3) × 10^−6^ M (n = 6) from the *K*_d_ value of 0 ^m^C-hp, respectively. The *Δ*G values increase to −35.3 ± 0.3 kJ · mol^−1^ (1 ^m^C-hp) and −32.2 ± 0.2 kJ · mol^−1^ (2 ^m^C-hp). We also measured ^m^C-G strength by the equilibrium dissociation constant *K*_d_ for hairpin formation in TMA-Cl. The *K*_d_ for ^m^C-G in KCl is 0.86 times the *K*_d_ for C-G, so without TMA insertion into ^m^C-G, the *K*_d_ for ^m^C-G in TMA-Cl should be 0.86 times the *K*_d_ for C-G, which is (2.85 ± 0.31) × 10^−7^ M. The true value of *K*_d_ in TMA-Cl is (6.37 ± 0.80) × 10^−7^ M, so the ^m^C-G strength is reduced 2.24 times. These results mean that the stability of methylated DNA is much lower than unmethylated DNA in 4 M TMA-Cl.

**Figure 2:**
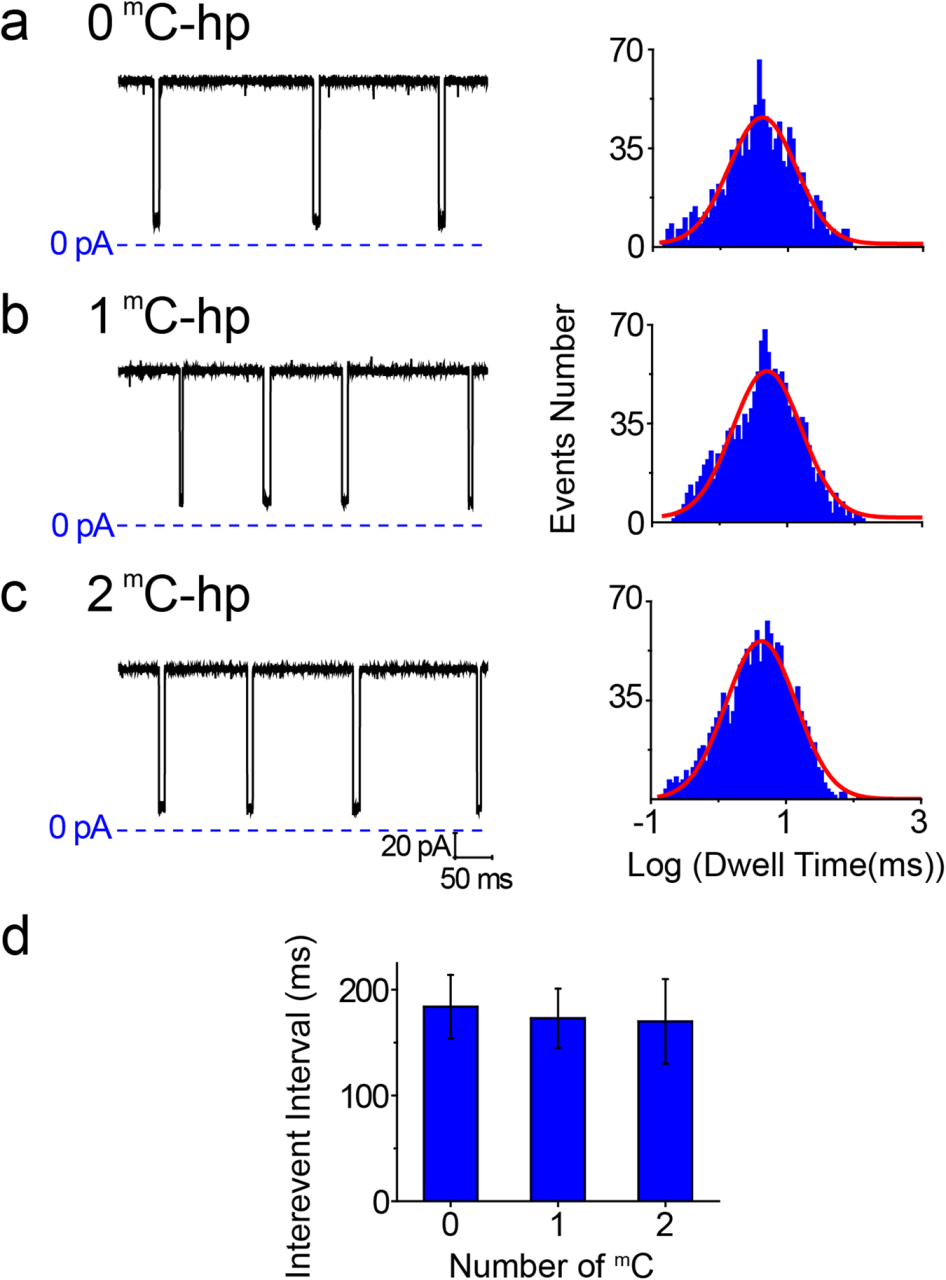
Protein nanopore sensing for DNA methylation in KCl. (**a**–**c**) The representative current traces (the left panel) of 0 ^m^C-hp (**a**), 1 ^m^C-hp (**b**) and 2 ^m^C-hp (**c**) in 1 M KCl. The corresponding histograms of the dwell time in log form are shown in the right panel. (**d**) Mean interevent interval, (n = 4) obtained from different methylated DNA in 1 M KCl. Other experimental conditions are the same as Fig. 1.

Using DNA fragments with the same sequence as above, we also tested their nanopore performance in KCl electrolyte. The results (see Fig. 2a–c) show that the dwell time values of all DNA fragments in KCl are much shorter than in TMA-Cl. The mean dwell time values for 0 ^m^C-hp, 1 ^m^C-hp and 2 ^m^C-hp were 4.57 ± 0.33 ms, 5.01 ± 0.24 ms 5.37 ± 0.79 ms, n = 4, respectively. This difference between the dwell time values of unmethylated and methylated DNA was less than 1 ms. In addition, there were no obvious differences between their mean interevent interval values (184 ± 30 ms for 0 ^m^C-hp, 173 ± 28 ms for 1 ^m^C-hp and 170 ± 40 ms for 2 ^m^C-hp, n = 4). The above data, including *K*_d_ and *Δ*G (Table S3), reveal that the KCl-filled nanopore had difficulty distinguishing the DNA.

### The effect of sequence context on detecting DNA methylation

**Figure 3:**
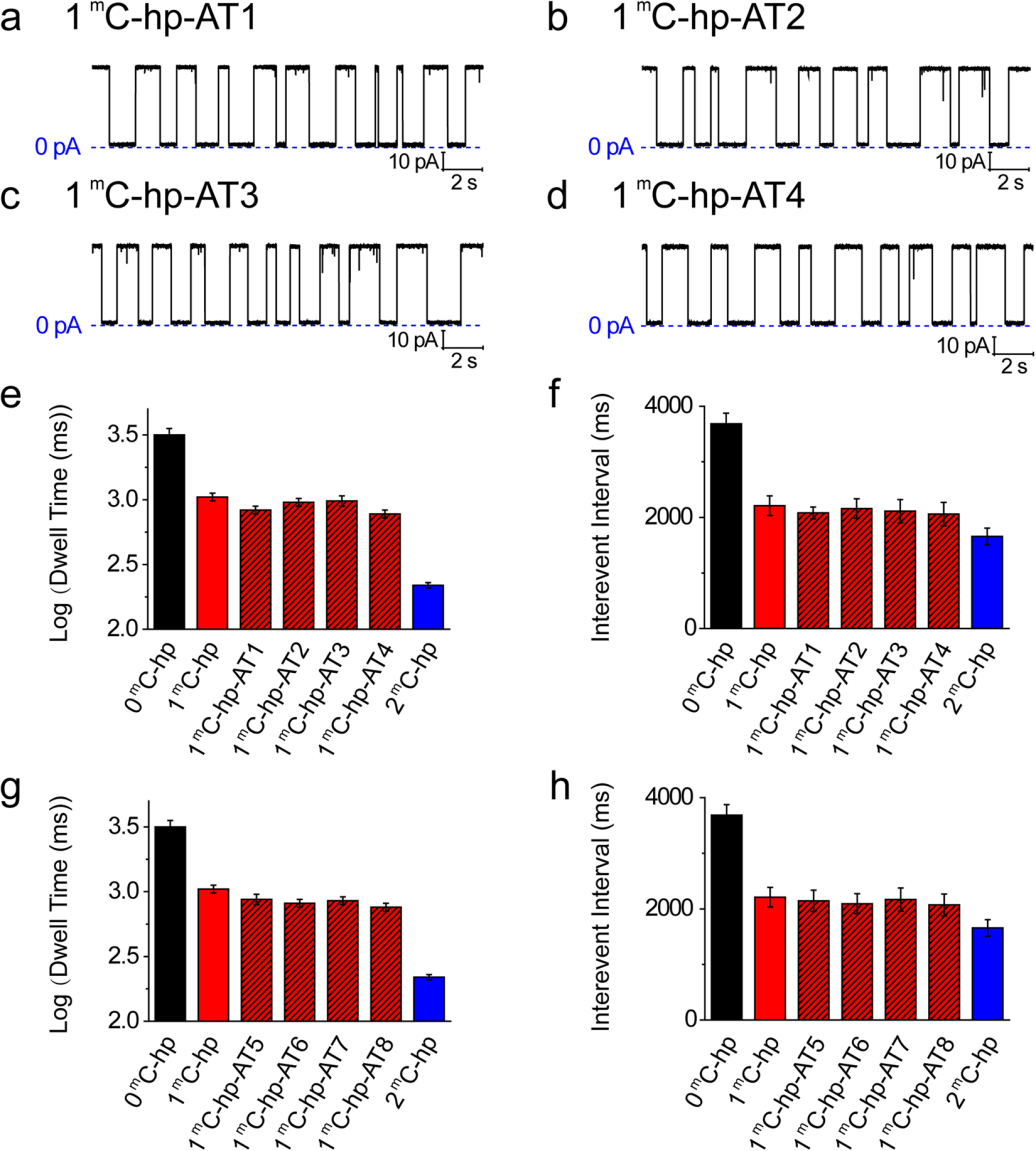
Effect of sequence context on distinguishing DNA methylation. (**a**–**d**) Current traces for 1 ^m^C-hp-AT1 (**a**), 1 ^m^C-hp-AT2 (**b**), 1 ^m^C-hp-AT3 (**c**), and 1 ^m^C-hp-AT4 (**d**) in 4 M TMA-Cl. (**e**,**f**) The peak value of dwell time (n = 4) in log-form histograms (**e**) and mean interevent interval (n = 4) of DNA hairpin translocation events (**f**) for 0 ^m^C-hp, 1 ^m^C-hp, 1 ^m^C-hp-AT1, 1 ^m^C-hp-AT2, 1 ^m^C-hp-AT3, 1 ^m^C-hp-AT4 and 2 ^m^C-hp. (**g**,**h**) The peak values of dwell time (n = 4) values in log form (**g**) and mean interevent interval (n = 4) values (**h**) for replaced 1 ^m^C-hps (1 ^m^C-hp-AT5, 1 ^m^C-hp-AT6, 1 ^m^C-hp-AT7 and 1 ^m^C-hp-AT8). The mean dwell times and mean interevent intervals were from single-channel recording, and signals were filtered at 2 kHz and sampled at 20 kHz. DNA (0.3 µM) was added into the *cis* chamber. The buffer was 10 mM Tris-HCl (pH 8.5).

The sequence context in which 5′-methylcytosine was embedded strongly affects the detection of DNA modification in KCl, as has been previously reported^42^. In this case, it would lead to difficulty or failure in detecting DNA methylation of random sequences. TMA ions have been shown to preferentially bind A-T base pairs^51, 59^. At high concentration, TMA has unique functions including enhancing the stability of A-T base pairs^60^ and eliminating the melting difference of A-T vs G-C base pairs in PCR, which do not result from fundamental changes in base stacking or base pairing^61–63^. Accordingly, we proposed that in a TMA-NP system, the sequence context might produce similar nanopore signals regardless of A-T or G-C base pairs. To test the hypothesis, we investigated the effect of adenine and thymine adjacent to and far from 5′-methylcytosine on distinguishing the methylation in TMA-NP. Four kinds of DNA hairpins were first designed, changing two base pairs of 1 ^m^C-hp from C-G to A-T immediately adjacent to 5′-methylcytosine (Table S1). For the replaced-DNA hairpins 1 ^m^C-hp-AT1, 1 ^m^C-hp-AT2, 1 ^m^C-hp-AT3 and 1 ^m^C-hp-AT4, the peak values of log (dwell time) in the histogram were 2.92 ± 0.03 (832 ± 59 ms), 2.98 ± 0.02 (955 ± 45 ms), 2.99 ± 0.03 (977 ± 70 ms) and 2.89 ± 0.03 (776 ± 56 ms), n = 4, respectively (Fig. 3e), and correspondingly the mean interevent intervals were 2145 ± 163 ms, 2094 ± 151 ms, 2167 ± 170 ms and 2072 ± 156 ms, n = 4, respectively (Fig. 3f). These data values were almost matched by 1 ^m^C-hp and obviously differed from 0 ^m^C-hp or 2 ^m^C-hp (Fig. 3e–f). In TMA-Cl, we then investigated the influence of the A-T base pairs far from 5′-methylcytosine. Four new DNA hairpins (Table S1, 1 ^m^C-hp-AT5~8) were tested in TMA-NP. The obtained peak values of log (dwell time) and the mean interevent interval were 2.94 ± 0.03 (871 ± 62 ms) and 2145 ± 168 ms for 1 ^m^C-hp-AT5, 2.91 ± 0.03 (813 ± 58 ms) and 2094 ± 151 ms for 1 ^m^C-hp-AT6, 2.93 ± 0.03 (851 ± 61 ms) and 2167 ± 151 ms for 1 ^m^C-hp-AT7, and 2.88 ± 0.03 (759 ± 54 ms) and 2072 ± 156 ms for 1 ^m^C-hp-AT8. These mean dwell time and interevent interval values showed no obvious differences, and were all very close to 1 ^m^C-hp (Fig. 3g,h). However, we noted that the dwell time values for the eight ^m^C-hps with A-T insertion were slightly smaller than that of 1 ^m^C-hp, but the maximum difference was only one-tenth of the difference between 0 ^m^C-hp and 1 ^m^C-hp. Therefore, TMA can remove the dependence on base sequence context in DNA strands in which the A-T bases are either close to or far away from the methylated point.

### Methylation Detection of P16 DNA gene fragment

**Figure 4:**
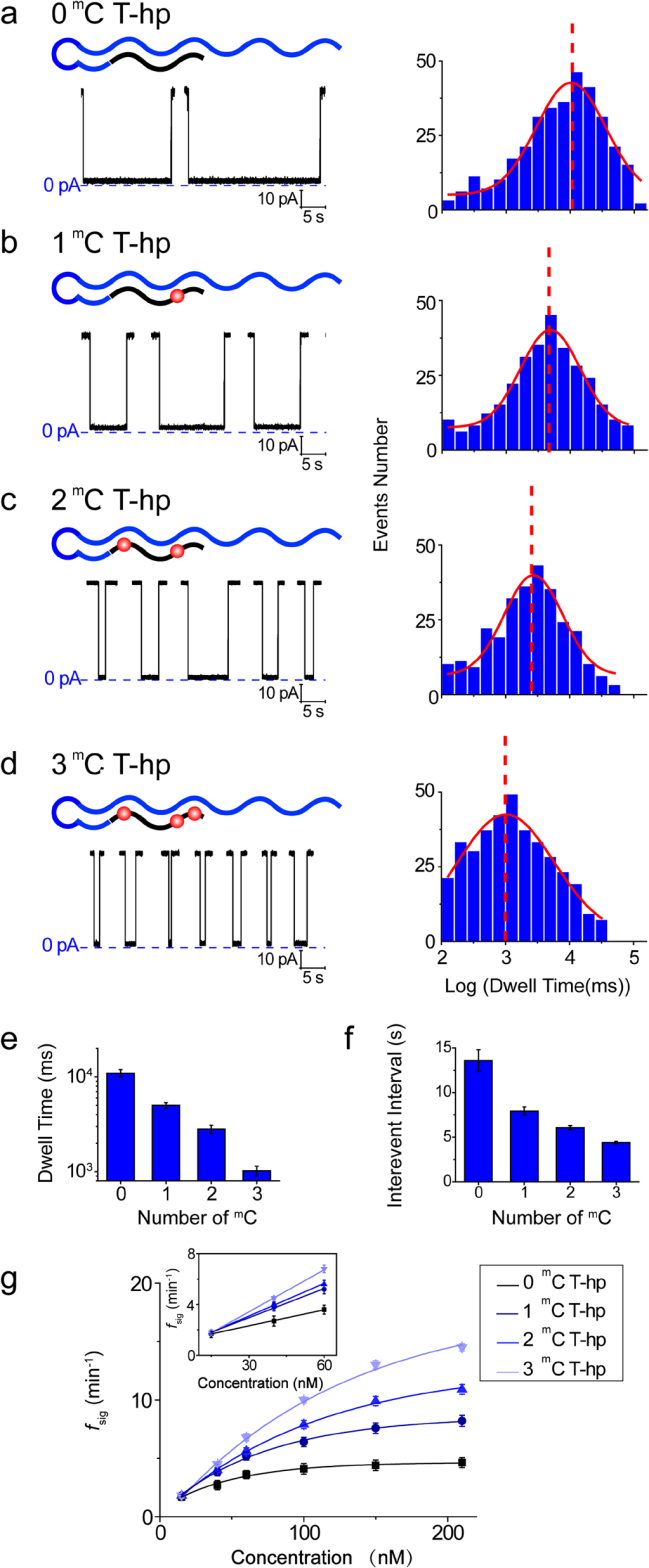
Methylation detection of the p16 DNA gene fragment. (**a**–**d**) Representative signal events and the histograms of target-probe DNA hybrids. A universal DNA probe (hp, blue colour) was used to identify and detect the methylation of different P16 gene fragments (black colour) containing zero 5′-methylcytosine (0 ^m^C T) (**a**), one 5′-methylcytosine (1 ^m^C T) (**b**), two 5′-methylcytosines (2 ^m^C T) (**c**), and three 5′-methylcytosines (3 ^m^C T) (**d**). Red represents the methylated points. (**e**,**f**) The peak values of log (dwell time) histogram (**e**) and mean interevent interval of hybrid DNA translocation events (**f**) for 0 ^m^C T-hp, 1 ^m^C T-hp, 2 ^m^C T-hp and 3 ^m^C T-hp. (**g**) *f*_sig_ shows the concentration dependence of target DNA from 15 to 210 nM. Inset: the plot of *f*_sig_ as a function of target DNA from 15 to 60 nM. Experimental conditions are the same as Fig. 1 except the applied voltage was +60 mV.

The tumour suppressor gene p16 (CDKN2A) is an important factor with widespread contribution to disruptions of the *cyclinD*-*Rb* cell cycle control pathway^64^. Methylation of p16 is strongly related to many tumour types, including colorectal, lung, and breast carcinomas^65–67^. We selected a 13-nt fragment from the antisense chain of the p16 gene promoter region within CpG island 176 (Chromosome 9:21, 994, 825–21, 994, 846) as a target sample to demonstrate the viability of TMA-NP in the detection of DNA methylation. The target fragments contained different methylated numbers and ^m^C distribution. To use a probe to detect the random points and distribution of the methylated bases, we designed a common probe consisting of a 5-base pair hairpin duplex, a 4-base loop, a poly (dC)_20_ “tail” and a 13-base strand that could specifically hybridize with the 13-nt fragment target (DNA sequences of target and probe are in Table S1). Target DNA fragments for unmethylated, one methylated, two methylated and three methylated points are expressed as 0 ^m^C T, 1 ^m^C T, 2 ^m^C T, 3 ^m^C T, respectively. When the hairpin probes (hp) were hybridized with target DNA, they formed hybrids 0 ^m^C T-hp, 1 ^m^C T-hp, 2 ^m^C T-hp and 3 ^m^C T-hp. The electrophoretic transport process of target-probe hybrid DNA through the α-HL nanopore is similar to that in Fig. 1a. However, at +40 mV, the DNA hybrids with an 18-base hairpin duplex had a slow translocation speed in 4 M TMA-Cl (>30 s). To save time, we used +60 mV to record single-channel events of these hybrids. With single-channel traces (Fig. 4a–d, left) and their histograms (Fig. 4a–d, right), the dwell time values obtained were 10965 ± 987 ms for 0 ^m^C T-hp, 5011 ± 358 ms for 1 ^m^C T-hp, 2818 ± 272 ms for 2 ^m^C T-hp and 1023 ± 122 ms for 3 ^m^C T-hp, n = 4. Comparing 3 ^m^C T-hp and 0 ^m^C T-hp, the translocation speed increased 10.72-fold, reflecting the high sensitivity of TMA-NP for methylated DNA. In the presence of only target DNA or probe DNA, they produced very short dwell times (0.47 ± 0.04 ms and 35.48 ± 2.67 ms, n = 4, respectively, Fig. S2), and thus did not affect the data collection of hybrids. The universal probe can be employed to identify and detect p16 DNA targets methylation, and different probes can be designed to detect their complementary DNA targets with random base methylation. In addition, the frequency of signature events *f*_sig_ can be used to quantify target DNA by the equation *f*_sig_ = 1/*τ*_on_ = *k*_on_ · C_DNA_, where *k*_on_ is the occurrence rate constant of signature events. We experimentally verified that *f*_sig_ is increased with an increase in target DNA concentration (C_DNA_) from 15 to 210 nM, as seen in Fig. 4g, which showed good linearity between the *f*_sig_ and target DNA concentration ranging from 10 to 60 nM. As the target DNA concentration increased above 100 nM, the increase in *f*_sig_ gradually becomes sublinear because nanopore is more likely to be occupied when another DNA molecule attempts to enter the nanopore.

An unmethylated p16 DNA gene fragment with an increased length of 3 bases (0 ^m^C T_L_) was also detected to distinguish between methylated ssDNA of a given length and unmethylated DNA of a longer length (Fig. S3). In addition to the previous probe, another probe (hp_L_) was designed with a prolonged 3 bases to completely pair with 0 ^m^C T_L_. 0 ^m^C T_L_ hybridized with these two probes (hp and hp_L_) are referred to as 0 ^m^C T_L_-hp_L_ (Fig. S3a) and 0 ^m^C T_L_-hp (Fig. S3b). The mean dwell time values of 0 ^m^C T_L_-hp_L_ and 0 ^m^C T_L_-hp were 56234 ± 8640 ms and 12022 ± 1160 ms, both longer than 1 ^m^C T-hp (5011 ± 358 ms), 2 ^m^C T-hp (2818 ± 272 ms) and 3 ^m^C T-hp (1023 ± 122 ms). The dwell time value of 0 ^m^C T_L_-hp was close to 0 ^m^C T-hp (10965 ± 987 ms), which can be attributed to the dwell time mainly depending on the hairpin length of hybrids, not the target length, and they increased 11.75-fold compared with 3 ^m^C T-hp. Therefore, an unmethylated p16 DNA gene fragment of longer length can be distinguished from a p16 DNA gene fragment of a given length.

## Discussion

The single-molecule technique developed in this work exhibits a number of advantages: first, it is the simplest and fastest method for detecting DNA methylation. The analytical process does not rely on bisulfite conversion, chemical modification or enzyme amplification; the results at the single-molecule level are directly reported in a few minutes by one simple step, recording TMA-NP current. Second, it is a precise and accurate method for detecting DNA methylation. In all recognition experiments for unmethylated, mono-methylated and multi-methylated DNA (total of 112 times), we obtained a zero-fault accuracy. The maximum error that occurred in the detecting sequence-dependent experiments was 7.8%. The high accuracy and precision largely originate from both the methyl-philic nature of TMA and TMA’s ability to remove base composition dependence.

In summary, we investigated nanopore biophysical characteristics of unmethylated and methylated DNA in TMA-Cl. We demonstrated that TMA can insert into the ^m^C-G bond and reduce its stability by 2.24 times. Interestingly, TMA can eliminate the stability difference of A-T vs G-C base pairs of DNA hairpin. In TMA-Cl, the base-pair stability order is A-T ≈ C-G > ^m^C-G, which is different from that in KCl (^m^C-G > C-G > A-T, see S8). Therefore, TMA-NP detection is independent upon the content and sequence of A, T, C, G, and only recognizes ^m^C. Given these findings, we successfully used a TMA-NP single-molecule tool to discriminate and detect P16 DNA gene fragments through a universal probe. The detection ability of TMA-NP for DNA methylation should have further broad applications.

## Methods

### Materials and reagents

Tetramethylammonium chloride was purchased from Sigma-Aldrich. DNA was purchased from AuGCT DNA-SYN Biotechnology Co., Ltd., (Beijing, China), and the DNA stock solutions were prepared in HPLC-grade water at a concentration of 100 μM. Hairpin DNA samples were heated in a 95 °C water bath for 5 min and cooled to room temperature over 3 h, and probe and target DNA samples were mixed at a 1:1 mol ratio, followed by heating in a 95 °C water bath for 5 min and then cooling to room temperature over 3 h. Lipid 1,2-diphytanoylphosphatidylcholine (DPhPC) was obtained from Avanti Polar Lipids (Alabaster, AL, USA). The 25 μm thick Teflon film was obtained from Goodfellow Inc. (Malvern, PA, USA). We used two buffers: 4 M TMA-Cl, and 1 M KCl, both prepared in HPLC-grade water and buffered with 10 mM Tris (pH 8.5). The WT-αHL monomers were first expressed in Escherichia coli BL-21 (DE3) pLysS and could be purified by size exclusion chromatography purification. Subsequently, they were assembled into homoheptamers by adding rabbit red cell membranes and incubating for 1~2 h. The heptamers were then purified by SDS-polyacrylamide gel electrophoresis and stored in aliquots at −80 °C.

### Planar bilayer recordings

A Teflon septum was used to divide the planar bilayer chamber into two compartments, cis and trans. A 1% w/v solution of DPhPC in pentane (Sigma-Aldrich) was spread across a 150 μm orifice in a Teflon partition by using the Montal-Mueller method. Preformed α-HL heptamers were added to the grounded cis compartment. Voltage was applied through a pair of Ag/AgCl electrodes. The DNA samples were added to the cis chamber compartment. The target and probe DNA were mixed in a 1:1 concentration proportion. The mixture was heated to 95 °C for 5 minutes, then gradually cooled to room temperature and stored at 4 °C until use. Currents were recorded with a patch clamp amplifier (Axopatch 200B, Axon Instruments, Foster City, CA, USA). The signal was filtered at a frequency of 5 kHz and sampled at a frequency of 20 kHz by a computer equipped with a Digidata 1440 A/D converter (Axon Instruments). All the recordings were conducted at 25 °C.

### Data analysis

Only the events with at least 70% of full blockage were included in the analysis to remove the collision events (DNA molecules colliding with the nanopore, but not fully translocated through the pore^35^). Data were analysed with the following software: pClamp 10.3 (Axon Instruments) and Origin 8.0. Mean dwell time values were obtained from the dwell time histograms by fitting the distributions to exponential functions.

## Electronic supplementary material

Supplementary information
