# Supplementary material for: Fast and precise detection of DNA methylation with tetramethylammonium-filled nanopore
Source: Sci Rep. 2017 Mar 15;7:183. doi: 10.1038/s41598-017-00317-2 (PMC5428259; doi:10.1038/s41598-017-00317-2)
Supplement: Supplementary file 1 — Supplementary information [file 41598_2017_317_MOESM1_ESM.doc]

***Supporting Information***

**Fast and precise detection of DNA methylation with tetramethylammonium-filled nanopore**

Ying Wang^1^, Yani Zhang^2^, Yanli Guo^1^ and Xiao-feng Kang*^,1^

^1^Key Laboratory of Synthetic and Natural Functional Molecular Chemistry, College of Chemistry & Materials Science, Northwest University, Xi'’an 710069, P. R. China

^2^College of Life Sciences, Northwest University, Xi’an 710069, P. R. China

*Corresponding author: Prof. Xiao-feng Kang

College of Chemistry & Materials Science

Northwest University, Xi'an 710069, P. R. China

Fax: +86-029-88302604;

Tel: +86-029-88302604;

E-mail: kangxf@nwu.edu.cn

**Contents**

1. Sequence of hairpin, probe and target DNA used in this study (Table S1).
2. Voltage (*V*) dependences of mean dwell time (*τ*_off_) (See Figure S1).
3. Data analysis of Kramers and Meller’s nanopore model.
4. Comparison of the kinetics and thermodynamics of three hairpin DNA in 4 M TMA-Cl (See Table S2).
5. Comparison of the kinetics and thermodynamics of three hairpin DNA in 1 M KCl ( See Table S3).
6. Current traces and corresponding histograms of unhybridized probe and target DNA in TMA-Cl (See Figure S2).
7. Methylation detection of p16 DNA gene fragment of different lengths (See Figure S3).
8. Comparison the stability of base-pair in KCl and TMA-Cl.

**1. Table S1. DNA Sequences used in this study***

| **DNA** | **sequence** |
| --- | --- |
| 0^m^C-hp | 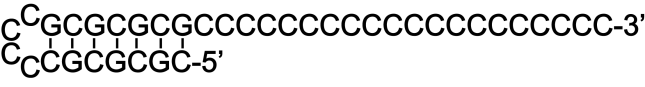 |
| 1^m^C-hp | 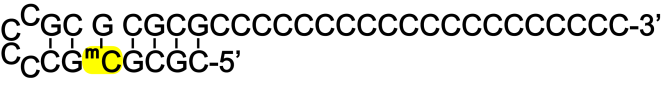 |
| 2^m^C-hp | 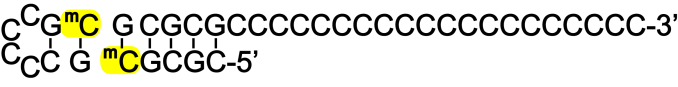 |
| 1^m^C-hp-AT1 | 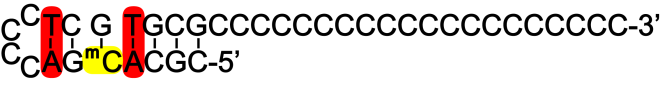 |
| 1^m^C-hp-AT2 | 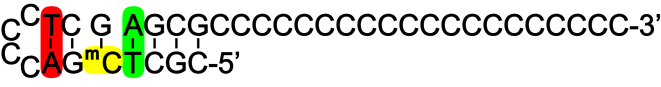 |
| 1^m^C-hp-AT3 | 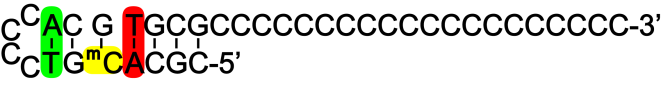 |
| 1^m^C-hp-AT4 | 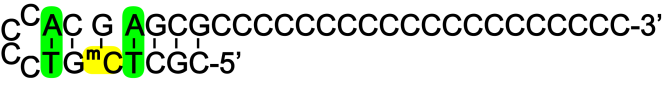 |
| 1^m^C-hp-AT5 | 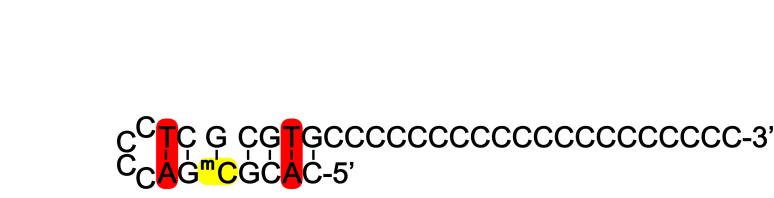 |
| 1^m^C-hp-AT6 | 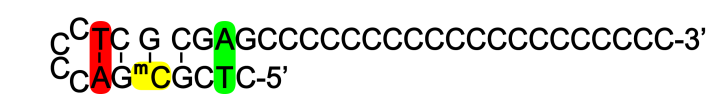 |
| 1^m^C-hp-AT7 | 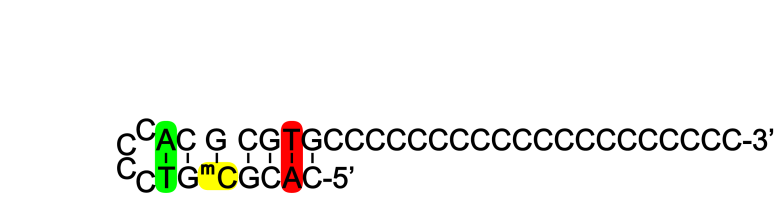 |
| 1^m^C-hp-AT8 | 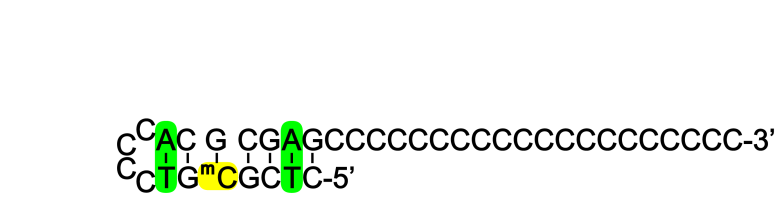 |
| probe | 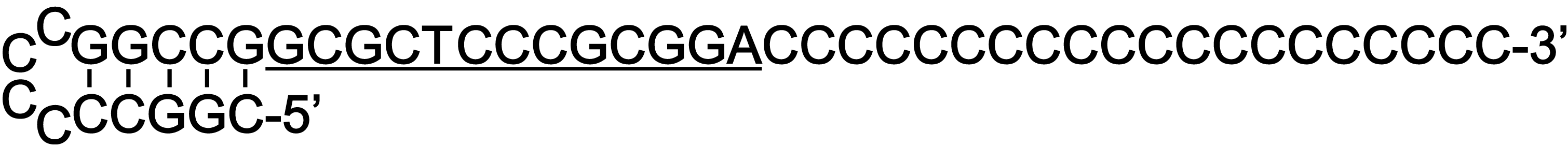 |
| 0^m^C Target | 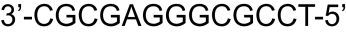 |
| 1^m^C Target | 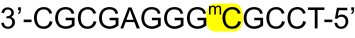 |
| 2^m^C Target | 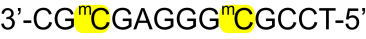 |
| 3^m^C Target | 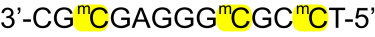 |

* 5'-methylcytosine was marked in yellow shadow. The replaced C-G base pairs in 1 ^m^C-hp were marked in red and green. The underlined sequence in probe was complementary to target DNA.

**2. Voltage-dependent curves of mean dwell time**


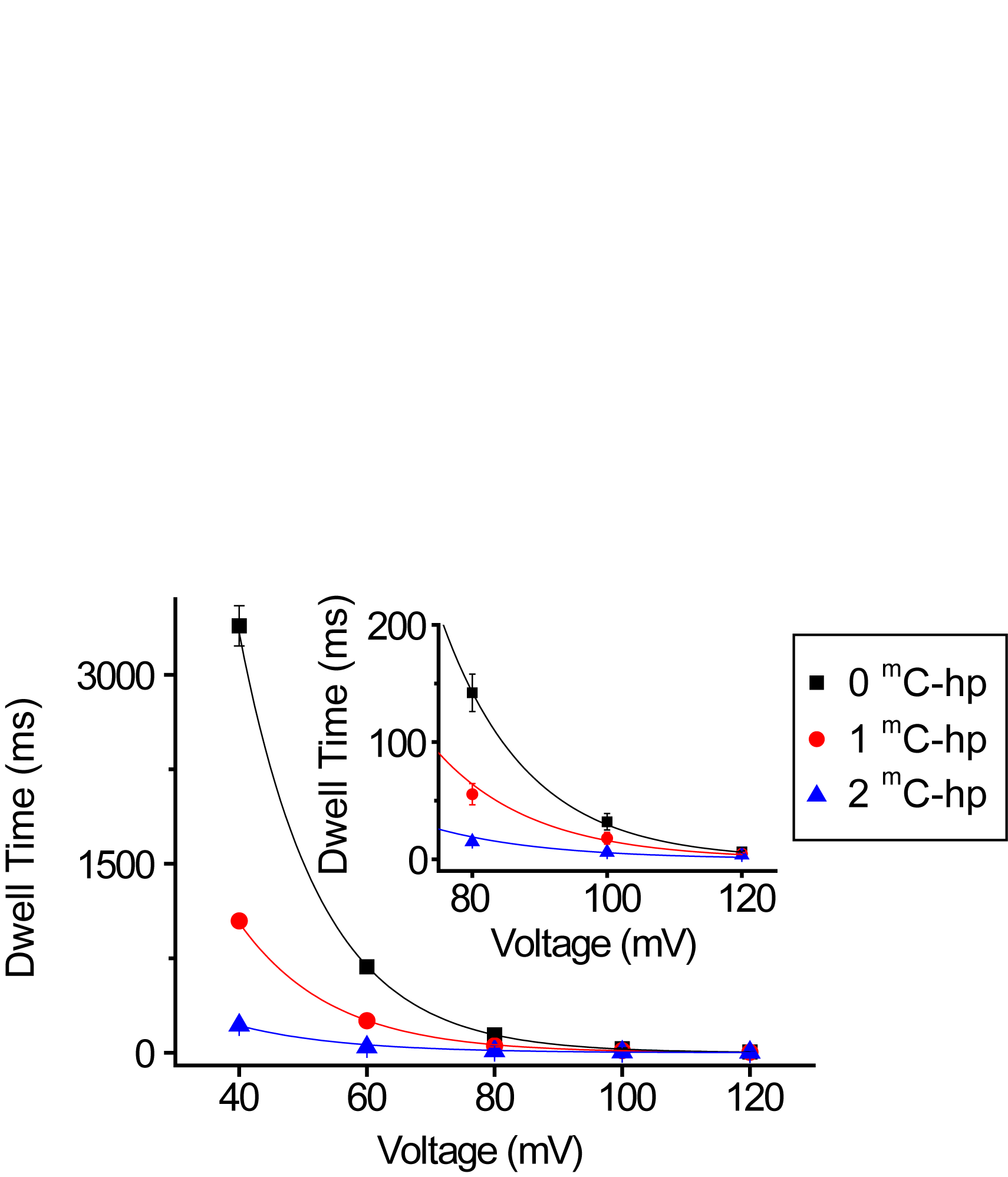


**Figure S1.** Voltage (*V*) dependences of mean dwell time (*τ*_off_) for 0 ^m^C-hp, 1 ^m^C-hp and 2 ^m^C-hp in 4 M TMA-Cl from +40 mV to +120 mV. The V~*τ*_off_ curves in the range of 40 mV~120 mV were enlarged (Inset). The lines were the best ﬁt to single exponential equation: *τ*_off_=7.79×10^4^*e*^(-0.079^*^V^*) for 0 ^m^C-hp, *τ*_off_=1.66×10^4^*e* ^(-0.069^*^V^*) for 1 ^m^C-hp and *τ*_off_=2.45×10^3^*e*^(-0.060^*^V^*) for 2 ^m^C-hp. Dwell time values were obtained from the histograms of dwell time by ﬁtting the distributions to exponential functions. The recordings were made at 25 ºC with a positive potential on the trans side and the cis side at ground.

1. **Data analysis of Kramers and Meller’s nanopore model**

According to Meller nanopore model^1^ developed on the basis of Kramers rate model^2^, nanopore unzipping kinetic of individual DNA hairpins with single-stranded overhang is an exponential voltage-dependent function:

*τ*_off_ = *Ae*(*^E^*^b-^*^Q^*^eff^ *^V^*)/*^k^*^B^*^T^* (1)

where *τ*_off_ is translocation time, *V* is applied voltage, *E*_b_ is the energy barrier for dissociation of the hairpin, *Q*_eff_ is the effective charge, -*Q*_eff_*V* is the reduction in the energy barrier due to the electric field, *K*_B_ is Boltzmann’s constant (1.38×10^-23^ J/K), T is temperature.

When applied voltage (*V*) is zero, zero voltage translocation time (or zero voltage transition time) *τ*_o_ = *Ae^E^*^b/^*^k^*^B^*^T^*. The experimental data of *τ*_off_ at different voltages are shown in Figure S1. All *τ*_off_ ~V curves present best ﬁt to single exponential equation: *τ*_off_=7.79×10^4^*e*^(-0.079^*^V^*) for 0 ^m^C-hp, *τ*_off_=1.66×10^4^*e*^(-0.069^*^V^*) for 1 ^m^C-hp and *τ*_off_=2.45×10^3^*e*^(-0.060^*^V^*) for 2 ^m^C-hp. When *V*=0, *τ*_o_ = *Ae^E^*^b/^*^k^*^B^*^T^*, thus *τ*_o_ (0 ^m^C-hp) =*Ae^E^*^b (0) /^*^k^*^B^*^T^*=7.79×10^4^ ms, *τ*_o_ (1 ^m^C-hp) = *Ae^E^*^b (1) /^*^k^*^B^*^T^*=1.66×10^4^ ms，*τ*_o_ (2 ^m^C-hp) = *Ae^E^*^b (2) /^*^k^*^B^*^T^*= 2.45×10^3^ ms.

By *τ*_o_ (0 ^m^C-hp)/*τ*_o_ (1 ^m^C-hp) and *τ*_o_ (1 ^m^C-hp)/*τ*_o_ (2 ^m^C-hp), the energy barrier differences are derived: *E*_b_(0)-*E*_b_(1)=1.55 J, *E*_b_(0)-*E*_b_(2)=2.05 J, displaying the ability of DNA methylation to reduce the energy barrier for dissociation of the hairpin.

Comparing *Q*_eff_ /*K*_B_*T* in equation 1 with the single exponential fit equations for 0 ^m^C-hp, 1 ^m^C-hp and 2 ^m^C-hp respectively, we estimated each effective charge (*Q*_eff_) in the pore: *Q*_eff_ (0 ^m^C-hp) = 2.03*e*, *Q*_eff_ (1 ^m^C-hp) = 1.77*e* and *Q*_eff_ (2 ^m^C-hp) = 1.54*e*. These effective charge values further verified the insertion of TMA^+^ into methylcytosine-guanine (^m^C-G) bond to offset DNA charges.

1. **Table S2 Comparison of the kinetics and thermodynamics of three hairpin DNA in 4 M TMA-Cl^α^**

| **DNA** | ***τ*_off_**  **[ms]** | ***τ*_on_**  **[ms]** | ***k*_on_**  **[10^6^ M^-1^·s^-1^]** | ***k*_off_**  **[10^-1^ s^-1^]** | ***K*_d_**  **[10^-7^ M]** | ***△*G *^b^***  **[kJ·mol^-1^]** |
| --- | --- | --- | --- | --- | --- | --- |
| 0 ^m^C-hp | 3388±160 | 3758±191 | 0.89±0.05 | 2.95±0.14 | 3.31±0.36 | -37.0±0.3 |
| 1 ^m^C-hp | 1047±49 | 2216±177 | 1.50±0.12 | 9.55±0.43 | 6.37±0.80 | -35.3±0.3 |
| 2 ^m^C-hp | 219±6 | 1657±150 | 2.01±0.16 | 45.7±1.3 | 22.7±2.9 | -32.2±0.2 |

^α^ Applied potential = +40 mV. *^b^* The values are for 25±0.5°C.

1. **Table S3 Comparison of the kinetics and thermodynamics of three hairpin DNA in 1 M KCl^α^**

| **DNA** | ***τ*_off_**  **[ms]** | ***τ*_on_**  **[ms]** | ***k*_on_**  **[10^7^ M^-1^·s^-1^]** | ***k*_off_**  **[10^2^ s^-1^]** | ***K*_d_**  **[10^-5^ M]** | ***△*G *^b^***  **[kJ·mol^-1^]** |
| --- | --- | --- | --- | --- | --- | --- |
| 0 ^m^C-hp | 4.57±0.33 | 184±30 | 1.81±0.25 | 2.19±0.15 | 1.21±0.29 | -28.1±0.6 |
| 1 ^m^C-hp | 5.01±0.24 | 173±28 | 1.93±0.25 | 2.00±0.10 | 1.04±0.21 | -28.4±0.4 |
| 2 ^m^C-hp | 5.37±0.79 | 170±40 | 1.96±0.37 | 1.86±0.24 | 0.95±0.26 | -28.7±0.6 |

^α^ Applied potential = +120 mV. *^b^* The values are for 25±0.5°C.

1. **Figure S2: current traces and corresponding histograms of unhybridized probe and target DNA in TMA-Cl.**


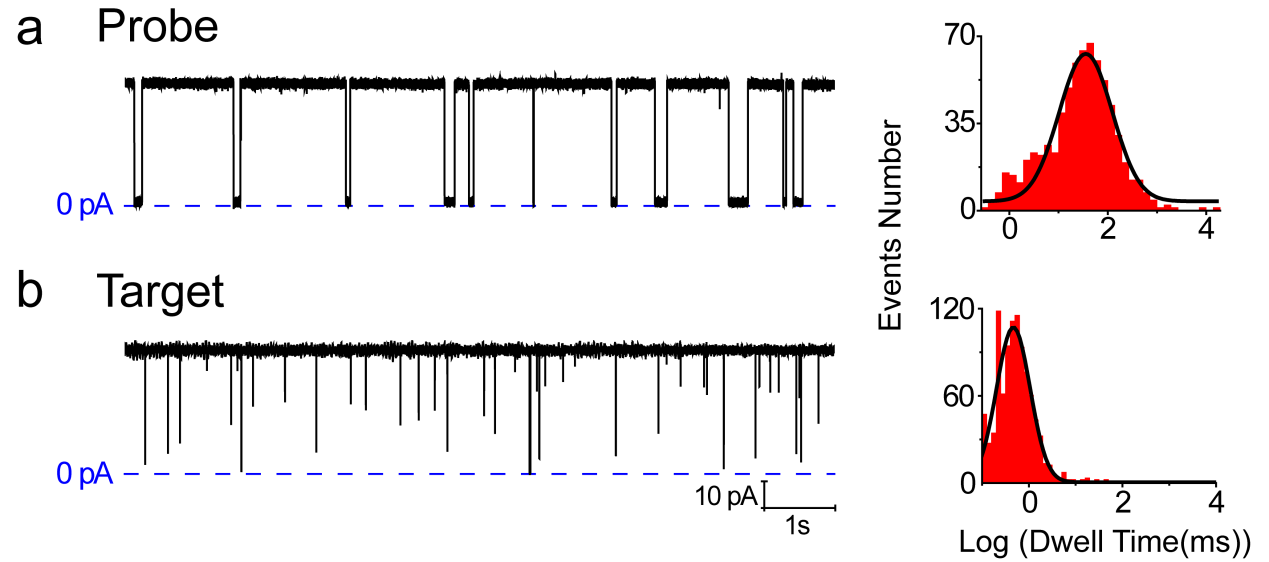


**Figure S2.** (a-b) The current traces (left) and corresponding histogram of the dwell time in Log form (right) for probe (a) and target DNA (b) in 4 M TMA-Cl. The signal was ﬁltered at 2 kHz and sampled at 20 kHz. The distribution of log dwell time was ﬁtted to a Gaussian function. The recordings were made at +40 mV, 25 ºC with a positive potential on the trans side and the cis side at ground.

1. **Figure S3: Methylation Detection of p16 DNA gene fragment of different lengths.**

**
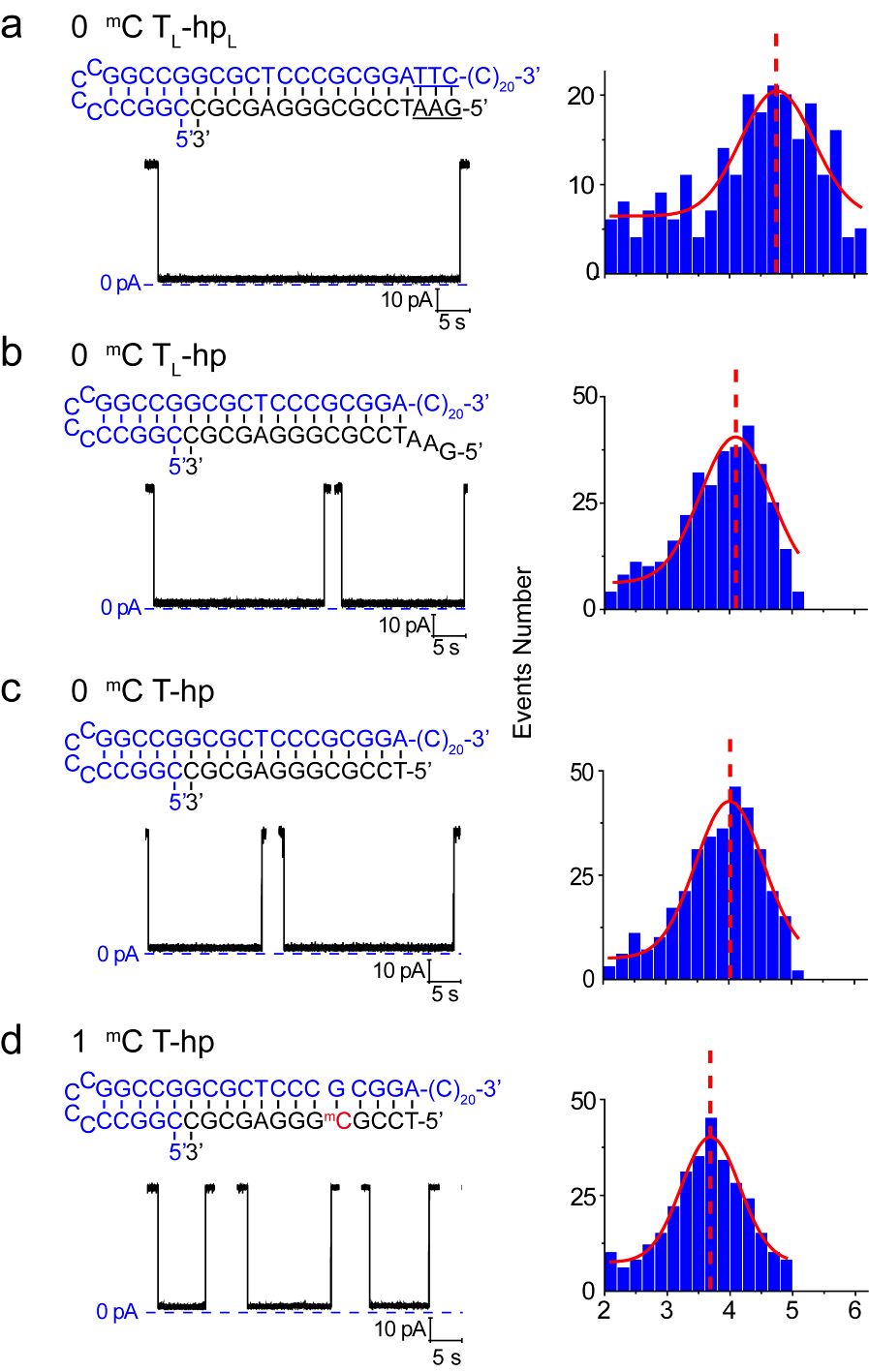
**

**Figure S3.** Methylation Detection of p16 DNA gene fragment of different lengths. (a-d) The sequences of target-probe DNA hybrids and their representative signal events and histograms. The DNA probe hp_L_ (a) and hp (b-d) (blue color) was used to identify and detect 16 bases (a-b) or 13 bases (c-d) unmethylated or methylated p16 gene fragments (black color) containing zero 5'-methylcytosine (0 ^m^C T) (a-c), one 5'-methylcytosine (1 ^m^C T) (d). Red color represents the methylated cytosine. Experimental conditions are the same as Figure 1 except the applied voltage +60mV.

1. **Comparison the stability of base-pair in KCl and TMA·Cl.**

**References**

1. Mathé J., Visram H., Viasnoff V. et al. Nanopore unzipping of individual DNA hairpin molecules. *Biophys. J.* **87**, 3205-3212(2004).
2. Bell, G. I. Models for the specific adhesion of cells to cells. *Science*, **200**, 618-627 (1978).
